# Assessing the virucidal activity of essential oils against feline calicivirus, a non-enveloped virus used as surrogate of norovirus

**DOI:** 10.1016/j.heliyon.2024.e30492

**Published:** 2024-04-29

**Authors:** Gianvito Lanave, Cristiana Catella, Alessia Catalano, Maria Stella Lucente, Francesco Pellegrini, Giuseppe Fracchiolla, Georgia Diakoudi, Jolanda Palmisani, Claudia Maria Trombetta, Vito Martella, Michele Camero

**Affiliations:** a Department of Veterinary Medicine, University of Bari Aldo Moro, 70010, Valenzano, Bari, Italy; b Department of Pharmacy-Drug Sciences, University of Bari Aldo Moro, 70125, Bari, Italy; c Department of Biosciences, Biotechnologies and Environment, University of Bari Aldo Moro, 70126, Bari, Italy; d Department of Molecular and Developmental Medicine, University of Siena, 53100, Siena, Italy

**Keywords:** Essential oils, Virucidal activity, Feline calicivirus, Norovirus, Surrogate

## Abstract

Norovirus (NoV) causes serious gastrointestinal disease worldwide and is regarded as an important foodborne pathogen. Due the difficulties of *in vitro* cultivation for human NoV, alternative caliciviruses (i.e., feline calicivirus, FCV, or murine NoV) have long been used as surrogates for *in vitro* assessment of the efficacy of antivirals. Essential oils (EOs) are natural compounds that have displayed antimicrobial and antioxidant properties.

We report *in vitro* the virucidal efficacy of four EOs, *Melissa officinalis* L. EO (MEO), *Thymus vulgaris* L. EO (TEO), *Rosmarinus officinalis* L. EO (REO), and *Salvia officinalis* L. EO (SEO) against FCV at different time contacts (10, 30 min, 1, 4 and 8 h). At the maximum non-cytotoxic concentration and at 10- and 100- fold concentrations over the cytotoxic threshold, the EOs did not decrease significantly FCV viral titers. However, MEO at 12,302.70 μg/mL exhibited a significant efficacy decreasing the viral titer by 0.75 log_10_ Tissue Culture Infectious Dose (TCID_50_)/50 μl after 10 min as compared to virus control.

In this study, virucidal activity of four EOs against FCV, was investigated. A lack of virucidal efficacy of TEO, REO and SEO at different compound concentrations and time contacts against FCV was observed whilst MEO was able to significantly decrease FCV titer.

## 1 Introduction

Noroviruses (NoVs) are single-stranded RNA viruses belonging to the *Caliciviridae* family. NoV was initially named as Norwalk virus, after the city in Ohio state where the virus was discovered [1]. Human NoVs (HNoVs) represent the second leading cause of viral acute gastroenteritis and represent a serious problem for food safety [2]. HNoV infections chiefly develop in medical centres, residential care homes, schools, and commercial and cruise ships [3].

Contamination by HNoV can occur during food production or preparation. When contaminated by water and food sources might represent a serious threat for disease transmission as they are often consumed raw [4,5].

Difficulties in NoV cultivation have caused many diagnostic challenges before the advent of biomolecular assays [1]. The shortfall of a duplicable NoV cultivation system has long hindered the evaluation of the antiviral activities of compounds/disinfectants against NoV. For this purpose, several cultivable viral models, including feline calicivirus (FCV), were used as HNoV surrogates [6]. Although surrogate viruses may not demonstrate firmly the efficacy of disinfectants against HNoV, these surrogates are regarded as useful models. Therefore, the effectiveness of sanitizers has long been evaluated employing cultivable HNoV surrogates [[7], [8], [9]]. Recently, B cells and human intestinal enteroids have been implemented to gather more precise information on HNoV properties [[10], [11], [12]].

The use of environmental disinfectants and personal hygiene products by staff handling foodstuffs is strongly recommended to control HNoV infections [13]. Since NoVs display resistance to sanitizers, it is crucial employing products with ascertained virucidal activity to hinder and contain virus proliferation [14]. There are several products that have been tested for the disinfection of surfaces and food against HNoV and hypochlorite and sodium bicarbonate and quaternary ammonium compounds proved to be very effective [[15], [16], [17], [18]].

Unfortunately, the organoleptic properties of many food products are affected by treatment with disinfectants at concentrations effective against NoV. Therefore, the food processing industries have a growing interest in non-chemical disinfectants able to inactivate HNoV without affecting the quality of food [19,20]. These natural products can be easily used on contaminated hands and surfaces due to their safety and ecological properties. Certain natural extracts, essential oils (EOs), erpenoids, lignans, alkaloids, and phenols (i.e., flavonoids, tannins, and coumarins), display antiinfective and antireplicative activities against viruses [20].

EOs are compounds of natural origin with antimicrobial and antioxidant activity. According to a chemical perspective, they consist of complex combinations of organic substances (monoterpenic and sesquiterpenic hydrocarbons and oxygenated substances i.e., phenols, alcohols, aldehydes, ethers, and ketones), in very different quantities [21,22]. They are usually characterized by 2 or 3 main components, present in high concentrations (from 20 to 70 %), and generally responsible for the biological action [23]. EOs are commonly used in mouthwashes [24], in disinfectant solutions [25], in aromatherapy and cosmetics for the preparation of creams, emulsions, soaps, perfumes [26].

Pellegrini et al., reported the virucidal efficacy of lemon EO (LEO) against FCV. LEO at the highest concentration decreased FCV titer up to 1.25 log_10_ Tissue Culture Infectious Dose (TCID_50_)/50 μL after 8 h of time contact [27]. In the present study the *in vitro* virucidal activity of different EOs against FCV was evaluated.

## 2 Materials and methods

### 2.1 Analysis of EOs

*Melissa officinalis* L. EO (MEO), *Thymus vulgaris* L. EO (TEO), *Rosmarinus officinalis* L. EO (REO), and *Salvia officinalis* L. EO (SEO) were provided by Specchiasol Srl, (Bussolengo, VR, Italy), and were stored in a sealed brown glass vials at 4 °C. Solvents, standard mixture of C10–C40 *n*-alkanes, and standard compounds reported in Table 1, Table 2, Table 3, Table 4 were purchased from Supelco Sigma-Aldrich Srl (Milan, Italy).

**Table 1:** Standard compounds related to *Melissa officinalis* L. essential oil (MEO). The most representative compounds are in bold.

N	Components	LRI	AI	*Melissa Officinalis*	
				Area ±SEM	SI/MS
1	α-pinene	930	931	0.34 ± 0.04	96
2	camphene	952	952	0.5 ± 0.05	96
3	β-thujene	968	968	0.2 ± 0.01	94
4	β-pinene	982	980	0.7 ± 0.05	91
5	eucalyptol	1023	1023	1.2 ± 0.5	98
**6**	**limonene**	**1030**	**1032**	**4.3 ± 1**	**94**
7	4-nonanone	1052	1053	0.3 ± 0.01	91
8	β-linalool	1100	1101	1 ± 0.2	97
9	citronellale	1168	1170	0.5 ± 0.05	93
10	α-terpineol	1178	1179	0.3 ± 0.01	80
11	citronellol	1220	1221	0.3 ± 0.04	95
**12**	**citral**	**1240**	**1240**	**43 ± 3**	**96**
13	geraniolo	1253	1254	2 ± 1	97
14	α-cubebene	1347	1348	0.4 ± 0.02	98
15	eugenol	1358	1359	0.15 ± 0.01	93
16	β-ylangene	1367	1368	0.13 ± 0.01	93
17	α -copaene	1374	1375	1 ± 0.1	99
18	geranyl aceate	1384	1385	2 ± 0.1	91
19	β-elemene	1394	1394	0.2 ± 0.01	83
**20**	**caryophyllen**	**1415**	**1415**	**25 ± 1**	**99**
21	*trans*-isoeugenol	1426	1427	0.3 ± 0.01	95
22	α-bergamotene	1431	1430	0.2 ± 0.01	87
**23**	**humulene**	**1452**	**1451**	**4.4 ± 0.9**	**97**
24	alloaromadendrene	1458	1458	0.16 ± 0.01	90
25	α-amorphene	1483	1484	1 ± 0.1	97
26	α-farnesene	1509	1508	0.13 ± 0.01	93
**27**	**caryophyllene oxyde**	**1596**	**1592**	**2.2 ± 0.9**	**91**
	% Characterized	/	/	92	/

**Table 2:** Standard compounds related to *Thymus vulgaris* L. essential oil (TEO). The most representative compounds are in bold.

N	Components	LRI	AI	*Thymus vulgaris*	
				Area ±SEM	SI/MS
1	propanoic acid, ethyl ester	714	714	0.1 ± 0.04	86
2	α-tricyclene	915	919	0.13 ± 0.1	94
3	α-thujene	925	926	1.2 ± 0.4	97
4	α-pinene	930	931	1.8 ± 0.1	95
5	camphene	949	952	2 ± 0.7	96
6	1-octen-3-ol	974	975	0.4 ± 0.01	83
7	sabinene	977	977	0.7 ± 0.3	93
8	β-pinene	978	978	0.6 ± 0.03	94
9	β-myrcene	985	991	1.4 ± 0.2	86
10	α-phellandrene	1001	1003	0.15 ± 0.01	91
**11**	***p*-cymene**	**1024**	**1024**	**20 ± 2.5**	**95**
12	limonene	1033	1027	0.6 ± 0.01	91
13	eucalyptol	1023	1031	0.9 ± 0.07	99
14	*cis*-β-terpineol	1145	1147	0.13 ± 0.01	90
**15**	**γ-terpinene**	**1063**	**1059**	**9 ± 0.9**	**94**
16	α-terpinolene	1085	1089	0.12 ± 0.01	81
**17**	**β-linalool**	**1097**	**1098**	**4 ± 1**	**97**
18	camphor	1145	1146	1.7 ± 0.8	98
19	borneol	1166	1167	1.9 ± 0.8	97
20	terpinen-4-ol	1172	1174	1.9 ± 0.8	96
21	α-terpineol	1189	1190	0.12 ± 0.01	86
22	methyl thymol, ether	1235	1235	0.4 ± 0.2	90
23	isothymol methyl ether	NA	1244	0.4 ± 0.02	94
**24**	**thymol**	**1290**	**1290**	**47 ± 1.6**	**94**
25	caryophyllene	1417	1418	2 ± 0.9	99
26	caryophyllene oxide	1581	1592	0.6 ± 0.1	91
	% Characterized	/	/	99	/

**Table 3:** Standard compounds related to *Rosmarinus officinalis* L. essential oil (REO). The most representative compounds are in bold.

N	Components	LRI	AI	*Rosmarinus Officinalis* L.	
				Area ±SEM	SI/MS
1	Tricyclene	920	919	0.5 ± 0.1	96
**2**	**α-pinene**	**930**	**931**	**20 ± 2**	**97**
**3**	**camphene**	**952**	**952**	**8 ± 1**	**97**
**4**	**β-pinene**	**982**	**980**	**4 ± 0.3**	**97**
5	1-octen-3-ol	–	–	0.2 ± 0.04	90
6	3-octanone	–	–	0.3 ± 0.07	91
7	β-myrcene	990	991	2.2 ± 0.1	96
8	α-phellandrene	1002	1003	0.34 ± 0.1	95
9	α-terpinene	1014	1014	0.4 ± 0.1	98
10	3-carene	1015	1016	0.5 ± 0.1	96
11	*o*-cymene	1021	1021	3.5 ± 0.4	95
**12**	**eucalyptol**	**1023**	**1022**	**23 ± 2**	**99**
13	γ- terpinene	1062	1064	0.6 + 0.1	97
14	terpinolene	1083	1085	0.6 ± 0.1	98
15	β-linalool	1100	1101	1 ± 0.1	96
**16**	**camphor**	**1145**	**1146**	**20 ± 1**	**98**
**17**	**endo-borneol**	**1166**	**1167**	**4 ± 0.7**	**97**
18	terpinen-4-ol	1171	1171	0.8 ± 0.1	96
19	α-terpineol	1178	1179	2.5 ± 0.3	96
20	verbenone	1204	1204	1.8 ± 0.8	98
21	bornyl acetate	1288	1289	1.3 ± 0.2	98
22	ylangene	1367	1368	0.13 ± 0.02	97
23	caryophyllene	1415	1415	2.1 ± 0.5	99
24	humulene	1452	1451	0.5 ± 0.03	96
25	caryophyllene oxyde	1596	1592	0.2 ± 0.02	90
	% Characterized	/	/	98.4 %	/

**Table 4:** Standard compounds related to *Salvia officinalis* L. essential oil (SEO). The most representative compounds are in bold.

N	Components	LRI	AI	*Salvia Officinalis*	
				Area ±SEM	SI/MS
**1**	**α-pinene**	**930**	**931**	**9.5 ± 1**	**97**
**2**	**camphene**	**952**	**950**	**8.8 ± 1**	**97**
**3**	**β-pinene**	**982**	**981**	**8.4 ± 0.7**	**97**
4	β-myrcene	990	991	1.5 ± 0.4	96
**5**	**eucalyptol**	**1023**	**1022**	**29 ± 2**	**99**
6	γ- terpinene	1062	1064	0.60 + 0.1	97
7	isoterpinolene	1086	1087	0.13 ± 0.04	98
8	β-linalool	1100	1101	3.2 ± 0.8	96
9	camphor	1145	1146	23 ± 1	98
10	endo-borneol	1166	1167	2.5 ± 0.7	97
11	terpinen-4-ol	1171	1171	0.2 ± 0.04	96
12	α-terpineol	1178	1179	2 ± 0.3	91
13	γ- terpineol	1195	1198	0.4 ± 0.1	94
**14**	**linalyl acetate**	**1256**	**1258**	**5 ± 0.4**	**91**
15	bornyl acetate	1288	1289	2 ± 0.4	99
16	caryophyllene	1415	1415	1.5 ± 0.8	99
17	humelene	1452	1451	0.3 ± 0.04	95
	% Characterized	/	/	91 %	/

### 2.2 Gas Chromatography/Mass Spectrophotometry (GC/MS)

A solution 1:100 v/v in ethyl acetate of each EO was prepared and filtered, then 1 μL of each solution was injected into the GC/MS. Analyses of EOs were carried out on an Agilent 6890 N gas chromatograph hyphenated with a 5973 N mass spectrometer provided with HP-5 MS capillary column (J & W Scientific, Folsom), using the conditions reported in our previous work [28].

### 2.3 Compound identification

Compound identification was carried out comparing the Linear Retention Indices (LRIs) and Similarity Index of Mass Spectra (SI/MS) for the obtained peaks with the Arithmetic Index (AI) and the analogous data reported in the literature [29] and in the NIST 2017 Databases (NIST 17, 2017. Mass Spectral Library-NIST/EPA/NIH. Gaithersburg, USA: National Institute of Standards and Technology. Last access 09_2023), respectively. The LRI of each compound was calculated with an equation related to a homologous series of *n*-alkanes (C10–C40) under the same operating conditions, as previously described [30]. SI/MS were determined as previously reported [31,32].

### 2.4 Cells and virus

Crandell-Reese Feline kidney (CRFK) cells were mantained at 37 °C in a 5 % CO_2_ atmosphere in Dulbecco-MEM, as previously described [27]. The same medium was employed for the antiviral assays.

The FCV field strain 283/12 was maintained and titrated on CRFK cells displaying a titer of 10^8^TCID_50_/50 μl.

### 2.5 Cytotoxicity assay

The cytotoxicity of all EOs was evaluated using an *in vitro* toxicological test kit (Sigma-Aldrich Srl, Milan, Italy), based on 3-(4,5-dimethylthiazol-2yl)-2,5-diphenyltetrazolium bromide (XTT). The assay was performed in triplicate as previously described [27]. MEO was evaluated at 8670, 4335, 2167.5, 1083.75, 541.87, 270.93, 135.46, 67.73 μg/mL. TEO was tested at 8900, 4450, 2225, 1112.5, 556.25, 278.12, 139.06, 69.53 μg/mL. REO was analysed at 8660, 4330, 2165, 1082.5, 541.25, 270.62, 135.31, 67.65 μg/mL. SEO was determined at 8790, 4395, 2197.5, 1098.75, 549.37, 274.68, 137.34, 68.67 μg/mL.

In the study, untreated cells and cells treated with equivalent dilutions of DMSO without EOs were used as control and vehicle control, respectively. The percentage of cytotoxicity for each EO was calculated using the formula: % cytotoxicity = [(OD of control cells−OD of treated cells) × 100]/OD of control cells.

The maximum non-cytotoxic concentration was evaluated and considered as the concentration at which the viability of treated CrFK cells decreased to 20 % compared to control cells (CC_20_).

### 2.6 Virucidal activity assay

One-hundred μL of FCV with a titers of 10^8^TCID_50_/50 μl were treated with the different EOs (900 μL) at different concentrations at room temperature for different interval times (10 min, 30 min, 1 h, 4 h, and 8 h). The virucidal effects of EOs were assessed in triplicate at the maximum non-cytotoxic concentration (CC_20_) and 10- and 100-fold concentrations over the cytotoxic threshold. The different mixtures of virus-EOs and untreated virus, defined as control virus (CV) were titrated on CrFK cells. Since LEO (a molecule of the same class/topology as other EOs) showed virucidal activity *in vitro* in the same laboratory settings/conditions [27], LEO was tested in parallel and used as “positive control” of the experiments.

### 2.7 Viral titration

Ten-fold dilutions (up to 10^−8^) of each supernatant were subjected to titration in quadruplicates in 96-well plates including CrFK cells. The plates were mantained for 72 h at 37 °C in a 5 % CO_2_ in incubator. Viral titer of the different mixtures of virus-EOs and CV was assessed according to the cytopathic effect, calculated according to the Reed–Muench method [34] and expressed as TCID_50_/50 μL.

### 2.8 Data analysis

Data derived from GC/MS and virucidal assays were described as area % ± SEM and mean ± SD, respectively.

EOs concentrations were transformed in log_10_, and cytotoxicity assay results were assessed by a non-linear curve fitting. Moreover, a dose-response curve was produced through non-linear regression analysis to estimate goodness of fit. From the fitted dose response curves acquired in each experiment, CC_20_ was obtained.

The normality of the data (Shapiro-Wilk test) and the homogeneity of variances (Levene median test) were assessed. If both requirements were fulfilled, the effects of time intervals for each substance concentration were evaluated by One-way ANOVA, succeeded by Bonferroni test as a *post hoc* test. The statistical significance level was always set at 0.05. Statistical analyses were performed using GraphPad Prism v8.1.2 program Intuitive Software for Science, San Diego, CA, USA.

## 3 Results

### 3.1 Analytical details of EOs

Analyses of the EOs displayed complex combinations of compounds which are reported in Table 1, Table 2, Table 3, Table 4 Thirty compounds were evidenced in MEO, representing 92 % of the total. MEO mainly presented citral (43 %), caryophyllene (25 %), humulene (4.4 %) and limonene (4.3 %) (Table 1).

A total of 26 components of TEO composed 99 % of the total detected constituents. The main components were thymol (47 %), *p*-cymene (20 %), and γ-terpinene (9 %), β-linalool (4 %) (Table 2).

Twenty-five components of REO were characterized and accounted for 98 % with respect to the whole detected constituents. The main components were eucalyptol (23 %), α-pinene (20 %), camphor (20 %), camphene (8 %), β-pinene (4 %), endo-borneol (4 %) (Table 3).

A total of 17 components of SEO were identified and accounted for 91 % of the total detected constituents. The main components were eucalyptol (29 %), α-pinene (9.5 %), camphene (8.8 %), β-pinene (8.4 %), Lynalin acetate (5 %) (Table 4).

### 3.2 Cytotoxicity

Cytotoxicity of the EOs was obtained by microscopic evaluation of cell morphology and assessment of cell viability by the XTT assay after putting into contact the cells to different concentrations of the substances for 72 h. The intensity and variety of cellular morphological modifications (loss of cell monolayer, granulation, cytoplasmic vacuolization, lengthening and shrinkage of cell extensions, and darkening of cell borders) were dose dependent [33]. Cytotoxicity was evaluated by calculating the absorbance signal spectrophotometrically. In all assays, the presence of DMSO did not show any consequence on cells. Based on the adjusted dose-response curves, the CC_20_ value of the EOs were calculated. CC_20_ for MEO accounted for 123.02 μg/mL whilst TEO displayed a CC20 equal to 194.98 μg/mL. REO exhibited a CC_20_ equal to 346.73 μg/mL and CC_20_ of SEO was equivalent to 1096.48 μg/mL.

### 3.3 Virucidal activity

**Fig. 1:**
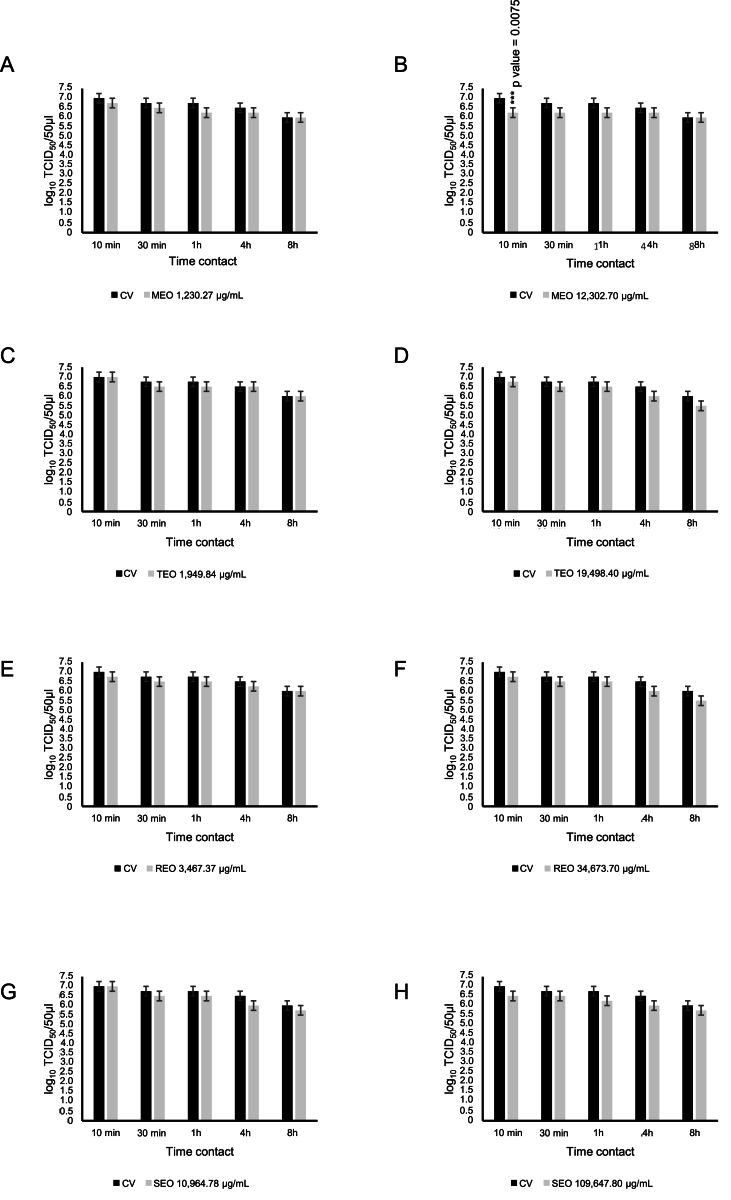
Virucidal effect of *Melissa officinalis* L. EO (MEO), *Thymus vulgaris* L. EO (TEO), *Rosmarinus officinalis* L. EO (REO), and *Salvia officinalis* L. EO (SEO) incubated with Feline Calicivirus (FCV) for 10 min, 30 min, 1 h, 4 h, and 8 h at room temperature and subsequently titrated in Crandell-Reese Feline Kidney (CrFK) cells. MEO was used at 1230.27 μg/mL (A) and 12,302.70 μg/mL (B), against FCV. TEO was tested at 1949.84 μg/mL (C) and 19,498.40 μg/mL (D), against FCV. REO was assessed at 3467.37 μg/mL (E) and 34,673.70 μg/mL (F), against FCV. SEO was used at 10,964.78 μg/mL (G) and 109,647.82 μg/mL (H), against FCV. Viral titers of different mixtures of virus-EOs and untreated virus, defined as control virus (CV) on CrFK cells at different time contacts are presented in the plot as mean of three different experiments. Viral titers of FCV were expressed as log_10_ TCID_50_/50 μL and plotted against different EOs at different time contacts. Significant p values are displayed. Bars in the figures indicate the means. Error bars indicate the standard deviation.

FCV was pre-treated with EOs at a maximum non-cytotoxic concentration (MEO at 123.02 μg/mL, TEO at 194.98 μg/mL, REO at 346.73 μg/mL and SEO at 1096.48 μg/mL). EOs, tested against FCV at CC_20_ and at 10- and 100- fold concentrations, over the cytotoxic threshold did not induce any significant reductions in viral titer (mean difference ranging from 0 to 0.50 log_10_ TCID_50_/50 μl) when compared to virus control at different time contacts (10, 30 min, 1, 4 and 8 h) and at room temperature (Fig. 1A-H).

However, MEO at 12,302.70 μg/mL displayed a significant inhibitory effect (p = 0.0075) against FCV decreasing the viral titer of 0.75 log_10_ TCID_50_/50 μl after 10 min as compared to virus control. Increasing the time contacts did not reveal significant effects of MEO against FCV (Fig. 1B).

One-way-ANOVA analysis evaluated on the viral titers of the EOs-treated FCV at different concentrations for different time contacts were related with the virus control, displaying a statistically significant effect in time contacts (F = 5.47, p = 0.0008) only for MEO when used at 12,302.70 μg/mL.

## 4 Discussion

Following the SARS-CoV-2 pandemic, the demand for chemical disinfectants for personal hygiene or surface cleaning has grown considerably. Unfortunately, the immoderate use of disinfectants (i.e., bleach and alcohol), used as antimicrobials, poses serious threats to humans and the environment [35]. Inhalation, ingestion, or skin absorption of alcohol in people below 12 years-old can induce intoxication with subsequent confusion, vomiting, and drowsiness. The excessive use of disinfectants and hand sanitizers increases the risk of contracting dermatitis due to alteration of the natural physiological barrier of the skin [36]. As previously observed for antibiotics and antivirals, microbial resistance has been reported also for disinfectants [36]. Furthermore, the chemical compounds present in disinfectants, if released improperly into the environment, are harmful because they are highly polluting for fauna, flora, and sea [35,36].

The growing awareness of the importance of health and hygiene and the concerns regarding the presence of viruses and bacteria in the environment have led to an increment in the demand for natural products with antimicrobial activities. Additionally, for many decades, governments worldwide have implemented policies to reduce or remove potentially hazardous chemicals in household cleaning and personal hygiene products [37].

The antimicrobial efficacy of various EOs against foodborne bacteria is widely reported [[38], [39], [40]]. Conversely, the antiviral activity of EOs against foodborne viruses is still under-investigated. Although the antiviral efficacy of EOs has been reported against enveloped RNA and DNA viruses [41], the effects of EOs against non-enveloped viruses remain to be elucidated. In previous studies, EOs have not been considered a valid alternative to control foodborne viruses in the food-processing industry [42], although the efficacy of EOs against non-enveloped RNA or DNA viruses (i.e., poliovirus, adenovirus, coxsackievirus) has been demonstrated [43].

In the present report, we evaluated the efficacy of four commercially available EOs (MEO, TEO, REO and SEO) against FCV. EOs were tested at the maximum non-cytotoxic concentration and at 10- and 100-fold over the cytotoxic threshold. Concentrations exceeding the cytotoxic threshold up to 100 times did not interfere with the reading of viral titers because the cytotoxic effects of the substance were observed only in the initial dilutions (10^1^ - 10^2^) of the titration. Starting from dilution 10^3^ the substances did not cause any effect reading of the titration plates.

The use of EOs at concentrations over the maximum non-cytotoxic threshold finds application in disinfection of surfaces. The application of EOs in food production could trigger deterioration and changes in organoleptic characteristics. Accordingly, it is preferable not to exceed with EOs concentrations due to the negative impacts of sharp and strong aroma not appreciated by consumers [44,45].

TEO, REO, and SEO, at all the concentrations tested in this study, did not induce any significant reductions in viral titer when compared to virus control at different time contacts (10, 30 min, 1, 4, and 8 h) and at room temperature. These results mirror what previously observed for other non-enveloped viruses which display a certain resistance to EOs [42,46,47].

However, MEO, in our study, at a 100-fold concentration over the maximum non-cytotoxic dose (12,302.70 μg/mL) displayed significant inhibitory effect against FCV decreasing the viral titer of 0.75 log_10_ TCID_50_/50 μl after 10 min compared to virus control. Surprisingly, the inhibitory effect of the substance on the virus did not show a time-dependent pattern, since extending the contact times did not increase the activity of the EO. MEO contained 27 distinct components, the main fractions of which were citral (43 %), caryophyllene (25 %), limonene (4.3 %), humulene (4.4 %) and, to a lesser extent, several terpenes and terpenoids. In a previous study, citral was moderately effective against Murine NoV type 1 (MNoV-1), producing significant reductions of up to 1.88 log_10_ TCID_50_/ml after 6 h [48]. Citral, a main constituent of lemongrass EO (over 50 %), also displayed antiviral activity against yellow fever virus and Herpes Simplex Virus type-1 [49,50]. Moreover, in a subsequent report, citral, despite being present to a lower concentration, was the most effective against MNoV-1 whilst other components of lemongrass EO (i.e., Ocimene, α-terpinolene, d -limonene, 1,4-cineole, and geraniol) did not display relevant effects on MNoV-1 inhibition [51]. Main components of MEO retrieved in this study should be investigated to reduce cytotoxicity of MEO and identify the active molecules against FCV. Despite the chemical composition of the solo EO constituents modify their exact mode of action and biological activity, presumably EOs biological activity strictly is based on the phytocomplex and is the consequence of unrelated and diverse mechanisms induced by synergic interactions with different cell targets.

Other EOs were also significantly effective against HNoV surrogates [52]. Oregano EO (OEO) induced a statistically significant decrease in MNoV-1 infectivity within 15 min of contact. Moreover, OEO affected the morphology of the viral particles [53]. However, in the OEO, citral was not identified among the components. The most representative substance of OEO was carvacrol which also proved to be active against MNoV-1 [53]. The EOs tested in our study did not have carvacrol among its components. LEO decreased FCV infectivity by 1.25 log_10_ TCID_50_/50 μL after 8 h of time contact [27] and was used as “positive control” in our experiments. The main constituent was represented by limonene (53 %), followed by β-pinene, γ-terpinene, citral, α-pinene and β-thujene. β-pinene along with α-phellandrene and limonene tested individually against MNoV-1 demonstrated significant virucidal effects [54]. In our study, although these fractions were retrieved in almost all the EOs tested, no remarkable effects were observed against FCV, likely due to their very low concentrations in the EOs.

TEO and one of its components, thymol, tested individually, demonstrated efficacy against HNoV surrogates in a dose-dependent manner, displaying a more significant reduction in FCV titers compared to MNoV-1 [55]. In our study, TEO, tested at different concentrations with respect to a previous study [55], did not display efficacy against FCV, despite the presence of thymol.

In REO and SEO, evaluated in our report, eucalyptol was observed in their composition ranging from 23 to 29 %. Both EOs were not effective against FCV. Although the antimicrobial efficacy of eucalyptol has been demonstrated [49,56,57], the data are still limited [58]. Moreover, efficacy of eucalyptol against caliciviruses has to be still elucidated.

Limited effects of EOs against HNoV surrogates were observed also in other reports. The lack of a relevant *in vitro* antiviral effects of hyssop and marjoram EOs against was also observed in MNoV-1 [42]. Viral infectivity of FCV strain F9 was reasonably decreased when the cell-free virions were put in contact for 1 h with 0.1 % of *A. princeps* var.*orientalis* EO (plaque reduction of about 48 %) before cell infection [59].

## 5 Conclusions

In conclusion, this study assessed the virucidal activities of different EOs against FCV, as a surrogate model useful to control HNoV contamination. EOs could be applied for food disinfection, although the aroma is required to be compatible with foods. EOs are able to interact either with the virus envelope causing its destruction or with the capsid proteins causing their disintegration with the subsequent loss of viral infectivity [53]. MEO, in our report, was able to affect the infectivity of FCV, a non-enveloped virus, notoriously resistant in the environment and to antimicrobials [60]. MEO was effective at the highest concentration tested overcoming the cytotoxic limit of 123.02 μg/mL, assessed in this study. Although, EOs are compounds of natural origin, side effects and contraindications should be considered in their applications for different uses. In our study, citral was the predominant fraction among the components of MEO. Citral is an acyclic monoterpene aldehyde largely applied to food, cosmetics, and beverages as a natural component for its intense lemon aroma and flavor [61]. For this component antimicrobial, antifungal, and antiparasitic properties have been reported [[62], [63], [64]].

EOs, being natural remedies rich in active components, present a very low toxicity threshold. It is therefore necessary to use caution during their application, since the limit between efficacy and toxicity sometimes is small [65].

**Declaration of Competing Interest**: The authors have declared that no competing interests exist.

## Funding

Not applicable.

## Institutional review board statement

The authors confirm that the ethical policies of the journal, as noted on the journal's author guidelines page, have been adhered to. Ethical approval was not required, and an ethical statement is not applicable as an *in vitro* study was performed.

## Informed consent statement

Not applicable.

## 8 Data availability statement

The data that support the findings of this study are described in the manuscript.

## CRediT authorship contribution statement

**Gianvito Lanave:** Writing – original draft, Visualization, Validation, Formal analysis, Data curation. **Cristiana Catella:** Investigation. **Alessia Catalano:** Supervision. **Maria Stella Lucente:** Investigation. **Francesco Pellegrini:** Software, Investigation, Data curation. **Giuseppe Fracchiolla:** Resources, Investigation. **Georgia Diakoudi:** Formal analysis. **Jolanda Palmisani:** Formal analysis, Data curation. **Claudia Maria Trombetta:** Supervision. **Vito Martella:** Writing – review & editing. **Michele Camero:** Writing – review & editing, Resources, Project administration, Methodology, Conceptualization.

## Declaration of Competing Interest

The authors declare that they have no known competing financial interests or personal relationships that could have appeared to influence the work reported in this paper.
